# Position statement and best practice recommendations on the imaging use of ultrasound from the European Society of Radiology ultrasound subcommittee

**DOI:** 10.1186/s13244-020-00919-x

**Published:** 2020-11-09

**Authors:** European Society of Radiology (ESR), Dirk-André Clevert, Christiane Nyhsen, Paolo Ricci, Paul S. Sidhu, Chrysa Tziakouri, Maija Radziņa, Vito Cantisani, Adrian P. Brady

**Affiliations:** European Society of Radiology (ESR), Am Gestade 1, 1010 Vienna, Austria

**Keywords:** Education, Ultrasound, Hygiene, Infection prevention, Medico-legal issues

## Abstract

This document summarises best practice recommendations for medical imaging use of ultrasound in Europe, representing the agreed consensus of experts from the Ultrasound Subcommittee of the European Society of Radiology (ESR), the European Union of Medical Specialists (UEMS) Section of Radiology, and the European Federation of Societies for Ultrasound in Medicine and Biology. Recommendations are given for education and training, equipment and its maintenance, documentation, hygiene and infection prevention, and medico-legal issues.

## Patient summary

Radiologists must take a leading role in training and governance of the use of ultrasound in clinical practice. Their concerns should be patient welfare and the safe, competent use of all imaging modalities for the benefit of patients. The standards, legislation, and best practice guidelines for carrying out ultrasound examinations should be the same for all professional users: both radiologists and non-radiologists.

Formal, documented, adequate, and continuous training in ultrasound is essential to ensure the quality of examinations. The focus must be on standards of education, training, equipment, and their use. This must include both lectures on fundamental principles and hands-on skills training. Furthermore, a documented briefing by a named person for the safe use of each device must be carried out.

Images must be stored on the device, then downloaded to a picture archive and communication system (PACS). The practitioner should document the indications and the findings of the ultrasound examination in a formal document as part of the patient’s permanent medical record and images should be easily retrievable.

Infection prevention and control measures must be implemented in order to prevent contamination. This includes rules on hand hygiene, decontamination of all external parts of ultrasound machines, regular deep cleaning of the entire ultrasound room’s surfaces and ancillary equipment.

Ultrasound equipment must be periodically assessed for image quality.

## Key points

- Adequate and continuous training in ultrasound is essential to provide quality examinations.
- Documentation of ultrasound images in a PACS system must be ensured.
- Hygienic measures must be implemented in order to prevent contamination.
- Radiologists must take a leading role in training and governance of the use of ultrasound in clinical practice.

## Introduction

In 2009, the eminent radiologists and ultrasound practitioners Derchi and Claudon articulated the position of US, claiming that US was an important strategic issue for radiology, with all possible solutions to maintaining a central role for radiology within US needed to be shared by the whole radiological community [1].

In 2010, the European Society of Radiology (ESR) published a Position Paper on Ultrasound [2], with the aim of defining the importance of ultrasound as a non-invasive imaging modality in the radiological world. The paper also demonstrated how ultrasound is often the cornerstone of patient diagnostic work up and stated that a Radiology Department must offer expert ultrasound on a 24/7 basis.

The paper highlighted the existence of ‘turf battles’ between radiologists and non-radiologists, which need to be overcome in the interests of patient care and improving the quality of ultrasound services within radiology and other clinical specialties. Above all, cooperation should be aimed at the development of training programs and guidelines for ultrasound imaging, designed to ensure high quality standards for examinations and proper access to services. At present, ultrasound equipment is available not only in the traditional sites (radiology departments), but also in many other clinical locations, indicative of a progressive decentralisation of general ultrasound onto clinical wards. Adherence to the same guidelines and the same operational standards (equipment, examination protocols, reporting, and archiving), regardless of where or by whom studies are performed, will help provide a comprehensive and uniform service.

Ten years later, and with collaboration between the ESR ultrasound Subcommittee and the Section of Radiology of the European Union of Medical Specialists (UEMS), it has been decided to update this position paper, producing a document that recommends appropriate standards for the use of ultrasound in Radiology, with particular reference to education and training, minimum requirements for ultrasound equipment, the impact of new tools in daily practice, practical aspects for the performance of ultrasound examinations (including image recording and storage, reporting etc.), equipment maintenance (including cleaning and disinfection), and medico-legal issues. The aim is to create a standard of practice for the use of ultrasound by Radiologists, which can also serve as a guideline for non-Radiologists.

Although radiologists are deeply involved in the field of ultrasound, this modality is extensively used by many other disciplines; cardiac, obstetric, and gynaecologic ultrasound are predominantly performed by non-radiology specialists. An increasing number of specialists are claiming ultrasound as a part of their everyday work, and the role of the radiologist in performing ultrasound is gradually being reduced. Radiologist involvement in ultrasound research is lessening, despite radiologists performing the majority of routine ultrasound diagnostic work. Furthermore, radiologists are disinclined to embrace new ultrasound techniques and developments e.g., contrast enhanced ultrasound, tissue elastography, which led to these techniques now being more readily embraced by non-radiology physicians. The newer younger generation of radiologists often regards ultrasound as less attractive than computed tomography (CT) or magnetic resonance (MR) imaging for a variety of reasons, including the necessity for a physical presence over a defined period of time with full attention, with direct patient contact and a perceived high volume of patients.

Ultrasound is pivotal in diagnostic practice, often representing the initial imaging examination of patients. In this current era where radiology is anxious to enhance the “visibility” of the specialty to the general public, the opportunity for direct radiologist contact with patients during ultrasound examinations emphasises radiology as a clinical discipline, and demonstrates that it is the radiologist, not the machine, that makes the diagnosis.

It can be argued that a non-radiologist physician experienced in ultrasound is better able to correlate ultrasound findings with the patient's clinical symptoms and signs. However, it is precisely the radiologist's greater imaging experience, and their familiarity with other imaging modalities, with an appreciation of imaging anatomy, which may complete the diagnostic process that allows the achievement of a more accurate diagnosis, ultimately rendering a precise answer to the clinical question. It should be fully appreciated that the radiologist is not a “technician” but a clinical medical specialist with imaging expertise, perfectly capable of correlating images with the patient's clinical picture.

This document is intended as a best practice checklist for the provision of an ultrasound service that is “fit-for-purpose”. Faced with growing discrepancies between ultrasound scanning methodologies, reporting processes for each body region, and the variable emphasis attributed by different operators to various aspects of ultrasound imaging, it was felt necessary to propose standardisation of ultrasound imaging examination technique, and to identify “standards” which anyone using the modality should observe.

## Education & Training

Ultrasound examination in the radiology department covers a range of different diseases, organs and vessels, performed in various clinical settings, ranging from urgent to routine and follow-up examinations. Additionally, it is used for guiding interventions, supporting intraoperative treatment, and monitoring patients in intensive care units. Furthermore, physicians from a wide range of specialties, including paediatrics, internal medicine, cardiology, emergency care medicine, gastroenterology, gynaecology, urology, and gastrointestinal surgery, perform ultrasound examinations [3].

There are several structural and operational differences among healthcare systems, appointment procedures, and training systems in the various European countries [4]. Since 2004, the European Federation of Societies for Ultrasound in Medicine and Biology (EFSUMB) has published a variety of guidelines, recommendations, and other policy documents concerning ultrasound [4]. This document takes into consideration the diversity of healthcare systems and demographic realities in Europe.

Presently, the national laws in each of the European Union (EU) Member States regulate professional activity within each country. In this context, it is important to build a comprehensive ultrasound core curriculum and training program that can be used in the individual countries according to the prevailing organisation and laws, and that complies with this legislation [5]. The ultrasound curriculum should constitute a uniform training program, approved by all European countries, which can be incorporated in pre-existing national training programs or serve as the basis for the development of new programs, according to national regulations.

The use of newer ultrasound technological developments has opened up new clinical applications. The most crucial point is education and subsequently preparation for updating of this acquired US knowledge [6, 7].

In many European medical school curricula, ultrasound imaging is already integrated as a core syllabus subject, to which up to 20 weeks may be devoted [8, 9]. Few universities have also advocated that ultrasound could be used not only for diagnostic imaging education, but also effectively in pre-graduate programs to teach medical anatomy and physiology [10–13].

Radiologists should be integral in the formulation of an ultrasound curriculum, as promoted by the Society of Radiologists in Ultrasound and the Alliance of Medical School Educators in Radiology (AMSER) [6]. In 2011, Hoppmann et al. detailed their first 4 years of experience in the School of Medicine at the University of South Carolina, where ultrasound was integrated in the curriculum [10]. Baltarowich et al. proposed in 2014 a two-fold curriculum organised as follows [14]:

- Pre-clinical: Utilisation of ultrasound to enhance students’ understanding of anatomy, physiology, and pathology.
- Clinical: Teaching students how to use ultrasound effectively as a problem-solving tool in the diagnosis of disease.

In Germany, for example, the necessary health requirements for professional ultrasound qualifications can be acquired by a combination of different options as follows [15]

- By professional qualifications in accordance with training regulations
- During a full-time 18 months period of ultrasound activity
- By attending ultrasound courses
- Through computer-assisted advanced training and ultrasound courses.

For example, the requirements for abdominal ultrasound include performing up to 400 examinations within a 6-month period.

Ultrasound training should be offered in a consecutive model. Basic courses should teach basic physical-technical knowledge, clinical indications, and basic knowledge of an ultrasound examination. Advanced courses should teach augmented knowledge of ultrasound diagnostics and examination technology. The advanced course may be replaced by a full-time period of clinical ultrasound activity of at least 4 weeks’ duration but must be carried out under the supervision of a qualified ultrasound physician. Final courses should complete knowledge and skills. The graduation course can be conducted as a complete ‘package’ course or as individual modules over time. In the final course examination, acquired ultrasound examinations will be scrutinised in both the written and image acquisition methods.

The basic course can be carried out as an interdisciplinary course. The advanced and the final courses must relate to the specific areas of application. There should be a period of at least 9 months between the basic and the final course of the student undertaking routine US scanning.

Lectures are often appropriate for teaching fundamental principles of ultrasound, but cannot replace the essential hands-on training that is critical for obtaining the skills necessary for handling a transducer and acquiring images simultaneously [9]. Modern educational media and material include simulation-based practical training, e-books, ‘*apps*’, interactive e-learning tools, examination technique videos, webinars, and case repositories (atlases). The challenge is to identify and maintain the quality of these educational tools, since there is significant potential for misinformation, as there is for any unregulated online educational modalities.

## Ultrasound equipment: general aspects, minimum requirements, and new tools

Requirements for equipment:

In locations under the jurisdiction of the German regulatory authority, ultrasound systems must comply with equipment safety, biosafety, and technical performance requirements. The minimum requirements are based on the level and class of application. For ultrasound systems that have been used for more than 24 months from the time of commissioning, a maintenance protocol must be available and submitted in addition to fulfilling the requirements. If a maintenance report cannot be submitted, an image-based acceptance test must be carried out to achieve approval. As part of the acceptance test, current image documentation must be submitted to assess the technical image quality performance of the US system [15, 16].

For each ultrasound device to be used, the practitioner must undergo a training for safe use held by a person who, based on their knowledge and practical experience, is suitable for instruction in the medical handling of the device. The briefing must be documented in writing, stating the name of the person giving the briefing [15].

Radiological equipment has a definite life cycle span, resulting in an unavoidable decrease of image quality over time, which renders equipment unusable after a certain time period. Each system should be used according to the regulations and recommendations of good quality technology standards – ultrasound equipment should be used actively up to 8 years and replaced afterwards [17].

Regulatory systems and oversight will differ in other countries, but these German standards represent a reasonable overview which can serve as a guideline elsewhere.

## Practical aspects for the performance of US examinations (including image storage & video recording, specific reporting)

The European Society of Radiology (ESR) Subcommittee on Ultrasound recognises that ultrasound examinations and reports are not always appropriately archived within hospital information systems [18]. Archiving of ultrasound images and reports is technically feasible, either as a separate archive dedicated solely to ultrasound, or, ideally, within the hospital Picture Archiving Computer System (PACS) [19, 20]. There may be difficulties in providing a network connection when ultrasound examinations are performed at the bedside or in an emergency setting, and large server capacities may be needed to store large amounts of data when videoclips or cine loops have to be recorded. However, the widespread use of wireless communication systems and continuous advances in archiving technology should overcome the current problems of data size and reviewing [21, 22]. Most modern ultrasound machines easily have sufficient memory to store images from a study until they can be downloaded to the PACS.

All ultrasound devices should be DICOM capable. In addition to these basic requirements, all the patients imaged should be registered on a DICOM worklist or RIS (Radiology Information System), as is common to all Radiology Departments, allowing a seamless record of the event. This ensures that the examination can be clearly assigned to the patient and that no information will be lost.

Regardless of where (or by whom) the ultrasound examination is undertaken, the data should be archived within a uniform, cross-facility software platform. The solution would mean that the Enterprise Picture Archive (EPA) would be used as a long-term archive and a uniform DICOM standard would be used at the hospital. This kind of software solution would enable the distribution of images within the facility as well as the reloading of ultrasound images into ultrasound or other imaging equipment, if necessary. Hence, via the clinical workplace system integration of the EPA and the integrated document management system, the examination and findings data should be available to all other departments involved in the patient’s management via a uniform access path.

A wireless LAN-based PACS device, first described 10 years ago by Lee et al., can help to reduce the time interval required for image storage and transfer to the main PACS, especially when it comes to transfer and storage of images obtained by portable modalities [23].

Ultrasound examination of any body part should be carried out according to a fixed scheme, as far as possible, although the order in which organs will be examined may vary; ultimately, the examination must be largely complete, and comparable with prior or subsequent examinations of the same area. Additional colour or power Doppler ultrasound examinations may be added, as appropriate. The practitioner performing the study must document the indication and the findings of the ultrasound examination on a formal document.

The medical documentation should contain:

I. patient identity (name and age)
II. investigator identification
III. date of examination (time if requested by local recommendations)
IV. indication for the examination
V. possible limitations of the examination due to scanning conditions etc.
VI. organ-specific description of findings, except for normal findings
VII. pathology characteristics
VIII. (suspected) diagnosis
IX. derived diagnostic and / or therapeutic consequences and / or suggestions for other investigations

This documentation describing the performance, findings, and outcome of the ultrasound study (the study report) must be filed as a formal part of the patient’s permanent medical record, and must be available for consultation to all authorised practitioners involved in the patient’s care.

In the case of normal findings, the archived images must show at least one or more suitable planes demonstrating the normal findings relevant to the clinical question. In case of pathological findings, the archived images should ideally show the abnormalities in two planes, or if this is not possible, clearly in one plane (only in B mode). The use of video clips can improve the visualisation of pathology, but at the expense of an increase in the data to be archived.

A question commonly encountered in clinical practice is whether radiologists should communicate the results of an ultrasound examination directly to the patient. There are several possible approaches to this issue, as discussed in the relevant literature [24]. Although this practice may cause some controversy, and variations in practice exist among departments and settings, it is an act of responsibility and respect for radiologists to play a role in communicating imaging results to patients, especially given that we will be dealing with them face to face, if performing the ultrasound study. In order to ensure optimal patient management, such matters should be discussed in advance with referring physicians and a concerted approach should be offered.

## Equipment maintenance

Regular maintenance and reliable equipment service are important in ensuring that a high-quality digital ultrasound system continues to function properly and retain its usefulness. Two different models for equipment maintenance are commonly followed. One is based on the hospital’s biomedical engineering staff (if they exist) performing yearly inspections ’in-house’. If this scenario is not available, maintenance may be provided under contract from the ultrasound machine vendor, or a third-party maintenance company. The ultrasound system is designated as medical equipment that contains several circuit boards, extensive service diagnostics, and complex operating software; it is recommended that only trained personnel service the machines. In addition to Original Equipment Manufacturer (OEM) service engineers, many ultrasound manufacturers offer service training for biomedical engineers. For budgeting purposes, it is recommended to take account of the system maintenance cost in the operational budget; the amount required will depend on multiple variables dependent of the level of risk aversion, and will possibly include software updates and accidental-damaged to transducers. For high risk averse customers, a comprehensive coverage service will include parts and labour replacement (including expensive items such as expensive transducers e.g. a trans-oesophageal transducer) for a fixed charge, but many OEM will offer a range of different service levels, tailored to a budget and risk.

To ensure technicians are qualified and effective, service providers need to train staff and have them certified with the OEM. To ensure speed of response and repair, the customers should investigate different levels of support/assist contracts with the OEM. These maintenance support agreements could include telephone remote support, trouble shooting, spare parts, and training to complement the skills of their own biomedical engineers to be more effective.

The basic maintenance should cover the following topics summarised in Table 1:

**Table 1:** Basic ultrasound maintenance

System Cleaning	
Transducer Care	
Printer and DVD Care	
Filter Cleaning/Replacement	
Basic Troubleshooting	
System Testing	

The advanced maintenance covers the following topics summarised in Table 2:

**Table 2:** Advanced ultrasound maintenance

System Service	
Transducer Test	
Parts Replacement	
Software Update Installation	

In Europe, ultrasound system checks are currently covered under the EN 60601-1 standard, which defines general requirements for the basic safety and essential performance of electrical systems connected to a power supply and intended for the diagnosis, treatment or monitoring of patients according to the manufacturer's instructions. This standard applies only to devices and systems that are in direct physical or electrical contact with the patient. A more uniform regulation will be used recently, and the main objective of the IEC 62353 is to reduce the complexity of the current system of Standards IEC 60601-1 (www.distler.de/en-62353, www.vde-verlag.de/normen/0700903/din-en-62353-vde-0751-1-2015-10.html) [25].

## Hygiene and infection prevention

In every clinical setting, particularly with high patient turnover and a large clinical team involved, it is important to establish and implement strict local hygiene and decontamination protocols. Standards need to be maintained to ensure patient safety for all ultrasound examinations and ultrasound guided interventions. Evidence of such protocols may need to be produced in litigation cases.

Contamination of ultrasound transducers is undeniably omnipresent [26–28]. The risk of cross infection secondary to ultrasound equipment has never been assessed by systematic research and the estimated actual risk is debated [29, 30]. Only a very limited number of adverse events have been published, but this should not be reassuring [31, 32]

National guidance and legislation regulating decontamination procedures vary throughout Europe. The European Society of Radiology ultrasound subcommittee (former ultrasound working group) found a great variance of practice throughout Europe and as a result has attempted to offer best practice recommendations to aspire to, which are briefly summarised below [26, 27].

Hygiene and decontamination protocols should be adapted to the local environment and clinical setting. They must include rules on hand hygiene (hands still being the primary source of infection transmission), decontamination of all parts of ultrasound machines, regular deep cleaning of the entire ultrasound room surfaces, as well as appropriate sharps and other waste disposal.

With the increasing use of smart phones by all members of staff, and marketing of the new “smart” ultrasound transducers that can be plugged into “phones”, no longer needing an independent ultrasound machine, it is essential to include decontamination of mobile phones or guidance on use restriction. Publications confirm worrying levels of phone contamination. According to Martina et al., > 99% of touchscreens showed partly multi-resistant bacteria and Simmonds et al. documented that despite high levels of contamination and staff awareness, only a minority (13%) disinfects their phones regularly [26, 27].

Viral phone contamination is more difficult to evaluate but studies also prove a contamination risk with viral pathogens. Pillet demonstrated viral contamination of almost 40% of examined mobile phones, predominantly from staff on paediatric wards [33]. Pathogens can survive a long time on inert surfaces, resulting in touch screens and other surfaces becoming a source of infection transmission for a prolonged period of time [34–37].

Summarised infection prevention recommendations include:

- Practitioner’s hands and ultrasound transducers used must be cleaned and disinfected before the first and after every subsequent patient contact. Should ultrasound gel or other dirt remain on the transducers, any disinfection agent is unable to penetrate and decontamination is incomplete; viable pathogens can persist in these circumstances.
- It is no longer acceptable to simply wipe off ultrasound gel between patients; practitioners cannot determine which “normal” microscopic flora patients may carry, which may pose a threat to the subsequent patient or which more virulent pathogens may be present on the patient’s skin. Accurate risk assessment of patients is difficult.
- Ultrasound transducer cables and keyboards/touchscreens of ultrasound machines (as well as all other clinical surfaces) should be very regularly fully decontaminated.
- Ultrasound gel bottles should not be kept open and upside down, in particular not in ultrasound bottle warmers. Pathogens may be present once the tip accidently touches the patient or other surfaces, and warmers may act as incubators. Single use bottles are preferable to refillable bottles.
- Should routine patients present with wounds or other skin pathology (any non-intact skin surface), ultrasound transducer covers are to be used with sterile gel inside and outside the cover as sheath micro-perforations cannot be excluded. This reduces potential infection transmission to the patient scanned as well as reducing transducer contamination, facilitating successful disinfection. Evidently, all ultrasound transducers need to be fully decontaminated after every patient contact, whether or not a transducer cover is used, due to micro-perforations and because of high contamination rates during cover removal.
- Low risk setting: After a routine ultrasound examination on intact skin, ultrasound transducer cleaning with subsequent low-level disinfection is sufficient.
- Intermediate to high risk setting: Following endo-cavity ultrasound examinations as well as after any interventional procedures (including ultrasound guided injections), high level disinfection is mandatory.
- All biopsy needles should be strictly single-use and re-usable needle guides should be avoided, as the thin bore is often not fully decontaminated.
- Single-use dedicated certified transducer covers must be used for endo-cavity ultrasound and all interventions.
- Sterile gel inside and outside transducer covers should be used for endo-cavity examinations and all interventions.

These recommendations need to be adapted to the actual clinical setting and should be reviewed regularly. Continuous staff training and quality assurance are essential. An initial investment and increasing ongoing consumable costs are likely to be necessary; cost implications therefore need to be considered but must not compromise patient safety. Technical developments with regards to automated decontamination facilities will undoubtedly aid in particular high-level disinfection and regular deep cleaning of ultrasound transducers, with costs hopefully decreasing. The aim should be to offer the best quality of care to all patients.

The full text publication “Infection prevention and control in ultrasound - best practice recommendations from the European Society of Radiology Ultrasound Working Group” [27] outlines further details as well as overview flow charts in the appendix.

## Medico-legal issues

In the era of modern imaging, medico-legal issues involving ultrasound are becoming more apparent, with the traditional radiology-based practice being eroded now that specialists other than radiologists are frequent users of ultrasound.

Amongst European countries, the legislative framework of ultrasound performance by non-radiologists varies significantly, ranging from strict legislation to a complete lack of guidelines.

In order to ensure optimal service delivery, and therefore avoid malpractice issues and unnecessary interventions, guidelines for proper education and performance standards of US have to be developed, with the aim of implementation of common policies among different countries.

Compared to other imaging modalities, medical use of ultrasound is highly operator-dependent and is fraught with scope for diagnostic error, the potential for which is magnified by the on-going development of more sophisticated equipment with extended applications [38].

A lack of formal documented training of ultrasound operators could leave the hospital / health organisation and the doctors performing ultrasound open to litigation if they are not adequately trained.

By comparison, radiologists are formally trained in the performance of ultrasound, shown by a survey in 2013 from the ESR Working Group on Ultrasound [18]. This survey assessed by what means diagnostic ultrasound was practiced and how training in ultrasound was organised in radiological departments of European hospitals. Questions were also aimed at evaluating the practice of ultrasound within both radiology and other hospital departments in order to understand the relationships among the different users of this technique. The results (91% of answers from teaching hospitals) highlighted that training is regarded as an art and is needed in order to learn the basics of scanning techniques, and that performance in an organ-oriented manner is the best way to learn how to integrate diagnostic ultrasound within the clinical context and also when combined with other imaging techniques.

A survey of national delegates by the UEMS-Section of Radiology in 2019, regarding “the performance of ultrasound by non-radiologists in European National Healthcare Systems”, highlighted the differences among European countries in terms of legislative variation and performance guidelines: None of the 19 European countries responding had specific legislation to regulate ultrasound performance by radiologists or non-radiologists; furthermore, 6 of the 19 had no specific educational requirements or guidelines [39].

These worrying results highlight the need for adoption of specific legislation and uniform guidelines regarding ultrasound performance by radiologists and non-radiologist medical specialists. In addition to this, updated guidelines should be adapted and frequently revised to include novel specialised imaging techniques, to ensure minimum quality requirements for performance of ultrasound examinations, aiming to preserve high-quality imaging services.

Overall, ultrasound medical users should be aware that they are legally accountable for their professional actions - including the reporting of ultrasound examinations, in all circumstances.

Several European countries allow ultrasound examinations to be performed by physicians only (Eastern Europe, Russia, France, Germany, Italy) In some Scandinavian countries, sonographers may perform ultrasound in Radiology departments, and midwifes perform obstetrical ultrasound. In Switzerland, there is a dual approach of having either ultrasound technicians supervised by radiologists (in the French-speaking part of the country) or radiologists performing ultrasound themselves (in the rest of the country). This is controlled by reimbursing only for ultrasound studies performed by physicians. For example, in Italy, the need to stardandise ultrasound medical practice led SIRM and SIUMB to establish a task force which produced a document called “Atto medico Ecografico” [40] defining the pre-requisites and the obligatory knowledge and activities for any medical doctor performing ultrasound. Some countries with state-funded healthcare systems and salaried employees (thus, individual study reimbursement does not arise) permit also non-physician ultrasound examinations (formally trained sonographers in UK, Israel, Ireland). However, considering the live scanning mode and operator dependency in the individual clinical setting, ESR strongly advocates ultrasound use by doctors, providing fast and accurate diagnosis using the multidimensional and pattern-based skills of radiologists or other medical specialists acquired during their speciality training.

There is also a substantial difference between conventional radiology department ultrasound and Point of Care Ultrasound (POCUS) [41]. For many clinical indications, ultrasound is the established first-line imaging modality worldwide [42]. Conventional ultrasound has been performed across multiple specialties for more than four decades. The equipment used is generally more sophisticated, hence more expensive and uses a wider variety of transducers across a wide range of imaging applications. Point-of-care ultrasound can be performed using less sophisticated and less expensive equipment, often portable or handheld devices. Point of Care ultrasound is designed to answer specific limited clinical questions and has become prevalent among various specialists for such limited uses, whereas diagnostic ultrasound as a key component of radiological practice allows for a more comprehensive and advanced clinical interpretation [41].

Another issue is whether the radiologist should perform diagnostic ultrasound scans and interventional procedures on patients who refer themselves directly to the ultrasound facility. This is particularly common in some private settings in subspecialties such as MSK and breast US, where imaging findings suggest the need for a therapeutic or diagnostic interventional procedure. While a danger of self-referral for financial gain exists in any scenario where a doctor stands to gain from initiating actions from which he/she will earn income, this is not confined to radiology. Most instances of self-referral in medicine arise from a sincere effort on the part of a doctor to utilise available tools to best effect. There is no reason to believe that conscientious radiologists will behave any differently from other clinical colleagues in terms of self-referral. Any rules that may be suggested must apply to all specialties, or none at all, and should be supported by evidence of their necessity.

Medico-legal issues that need to be addressed include:

- **Training Requirements, Certification, Competence, and Recertification in Ultrasound:**

**Non-Radiologists:**

- Minimum requirements and training within the specific specialty (e.g. Gastroenterology, Internal Medicine, Surgery, Urology) should be defined.
- Minimum requirements and training should be defined for non-specialised Medical Doctors, (i.e. definition of practice level, recognised clinical training and competency, etc.).
- Syllabus assignment for anatomical areas corresponding to their medical specialty.
- Duration of training in balance with ESR curriculum requirements [42] of ultrasound training for radiologists: from 3 to 8 months training period with subsequent regular hands-on sessions from 250 to 2000 studies within a 2-year period, or practical training should involve at least one ultrasound list per week (10 examinations) - varying pre-certification requirements among the European countries.
- Decision on what courses, seminars, and ultrasound examinations are needed for the appropriate training under the supervision of qualified trainers.
- Specification of the qualification which should be held by institutions and trainers.
- Coherent tests and hands-on examination preparation.
- Certificate examinations.

**Radiologists:**

- Radiologists are certified in ultrasound performance by obtaining their specialty certification, based on the ESR curriculum, derived from a minimum of 8-12 weeks basic training in ultrasound, with subsequent regular hands-on sessions integrated in all modules and subspecialty practical training.
- Within the field of Radiology, minimum requirements and training should be defined for those wishing to be granted subspecialty certification, such as: musculoskeletal, vascular, neurological, paediatric (including brain), endocrine, transcranial, neonatal, gastrointestinal, gynaecological ultrasonography, and the ability to perform advanced ultrasound-guided procedures. These requirements may include, among others, definition of practice level, recognised clinical training and competency, etc.

b) **Accreditation of the Training Program- Teaching Institutions:**
  National professional licensing bodies, or in their absence, a European association/society/organisation (UEMS, ESR), should provide a general program for accrediting teaching institutions. It is recommended that this accreditation process be valid for a limited time and renewed on a regular basis, according to national regulations.
  Institution accreditation for candidate training in ultrasonography performance should only be granted or renewed if the applying program documents have a minimum required annual activity of ultrasound imaging. The accrediting authority for each applying institution may define an agreed intermediate level of activity.
  The teaching program should be established within an institution, or a network of such institutions, with all the appropriate related specialties represented.
  The director of the training program should be certified according to national regulations and may have a senior academic appointment or a senior leading position in a non-profit training institution.
  Ultrasound equipment evaluation in training institutions.
c) **Definition of Criteria for Qualified Trainers and Examining Committees:**
  Examination Committee for certification/license in performing ultrasound after training should be selected by the relevant professional organisation, depending on the specialty.
  The Examination Committee should comprise at least three members, consisting of two doctors of the corresponding specialty that have permission to perform ultrasonography and one other competent individual (a radiologist practising ultrasound would be an ideal member).
  Leaders of the training team should be involved in examining committees.
  Basic requirements for teachers should be defined. This could reflect experience in a clinical subspecialty in a field which he/she will only teach (e.g. gastrointestinal, musculoskeletal, vascular, paediatric, etc.). Participation of radiologists with specialist expertise in ultrasound should be encouraged in training of non-radiologists.
  The number and type of ultrasound examinations performed by the trainer on a regular basis, both in emergency and inpatient hospital settings, should be defined. Ideally, a minimum number of cases annually should be a necessary criterion for continuation as a trainer.
d) **Evaluation of Trainees, Duration of Training-Examination, and Certification**
  Physicians must meet procedure volume requirements on the type(s) of accreditation for which the practice is applying.
  The program director, in consultation with the co-directors and faculty, will evaluate the qualification and progress of each candidate trainee. Evaluation includes assessment of the trainee's knowledge, technical skills, attitudes and interpersonal relationships, decision-making skills, and clinical management skills.
  The program director, in agreement with the co-directors, certifies the competence of trainees at completion of training.
  License certification for ultrasonography performance will be granted once the candidate has successfully met the aforementioned pre-requirements and following official examination. Limited number of pre-requirements will include amongst others, level of training, technical and medical competency, performance of a minimum number of ultrasound studies, etc.).
  The evaluation must be approved by a certified national board.
e) **Maintenance of Competence – Recertification.**
  Recertification should be based on a pre-organised network of accredited institutions/departments.
  At reaccreditation, all physicians who were previously granted licences for ultrasound performance must document a prespecified minimum number of ultrasound examinations corresponding to their area of expertise.
f) **Best clinical practices in ultrasound performance**
  Requirements regarding equipment;
  Infection prevention and control;
  Archiving and reporting of ultrasound images (i.e.: definition of minimum of images and videos required to be added, and issuance of formal reports).
g) **Financial compensation of ultrasound practice by non-Radiologists in the National Health Systems**
  Only those fulfilling the criteria mentioned above (formal licensing, limitation to specific fields of expertise, official reaccreditation, etc.) should be financially compensated for their medical act regarding ultrasonography use, subject to the legal and institutional form of reimbursement prevailing in each country.
  In case these criteria are not fulfilled, financial compensation should not be endorsed. In these circumstances, ultrasound examination will only be used as supplementary to clinical decision making and guidance. Financial compensation prerequisites will act as a driving force for appropriate training, thus safeguarding a minimum quality provision of ultrasound imaging.
  General practitioners/GPs working in National Health Systems in most counties are entitled to train and use ultrasound as a supplement to clinical decision making and guidance, but they are usually not financially compensated for this use of ultrasound.

## Safety

Ultrasound applications are considered safe for patients, according to the guidelines of the British Medical Ultrasound Society (BMUS) and European Federation of Societies for Ultrasound in Medicine and Biology (EFSUMB). However, depending on the application and device, thermal effects could theoretically occur in the tissues being scanned, particularly in the case of Doppler ultrasound [43].

Ultrasound education should include discussion of possible effects of ultrasound on human tissue, mainly through thermal and non-thermal (or mechanical) mechanisms [44]. These relate to tissue heating, cavitation and mechanical overload [45]. The “as low as reasonably achievable” (ALARA) principle [46] of potential thermal effect, as described by the thermal index (TI), and mechanical effect, described by the mechanical index (MI), should be included in ultrasound education, under the heading of safety.

## Closing Remarks and Outcomes

In most European countries, there is no legislative provision for limiting the performance of ultrasound to any specific group of medical doctors, or even to doctors in general. It is not likely that this position will change in the near future. As radiologists, our concerns should be patient welfare, and safe, competent use of all imaging modalities for the benefit of patients. Our focus must be on standards, of education, training, performance of studies, and the other elements of ultrasound practice outlined in this article.

The standards applied to imaging use of ultrasound should be the same for all users of the modality. As imaging professionals, radiologists have integrated knowledge of different imaging modalities and techniques which positions us well to act as arbiters of good ultrasound practice. Adoption of legislation and best practice guidelines for carrying out ultrasound examinations must be common for both radiologists and non-radiologist physicians. Success in harmonising ultrasound standards and practices will require cooperation among all interested parties, including UEMS, ESR, EFSUMB, and other specialty societies; consensus positions can then be proposed and advocated in a united manner, for future legal implementation.

## Data Availability

All data generated or analysed during this study are included in this published article.

## Abbreviations

AMSER: Alliance of Medical School Educators in Radiology
BMUS: British Medical Ultrasound Society
CT: computed tomography
EFSUMB: European Federation of Societies for Ultrasound in Medicine and Biology
EPA: Enterprise Picture Archive
ESR: European Society of Radiology
EU: European Union
MR: magnetic resonance
OEM: Original Equipment Manufacturer
PACS: Picture archive and communication system
POCUS: Point of Care Ultrasound
RIS: Radiology Information System
UEMS: European Union of Medical Specialists
